# Rat polymyxin b hemoperfusion model: preventive effect on renal tubular cell death in a rat cecal ligation and puncture model

**DOI:** 10.1186/cc14207

**Published:** 2015-03-16

**Authors:** T Masuda, C Mitaka, M Khin Hnin Si, K Kido, Y Qi, T Uchida, S Abe, T Miyasho, M Tomita

**Affiliations:** 1 Tokyo Medical and Dental University, Tokyo, Japan; 2 Tokyo Medical and Dental University Graduate School, Tokyo, Japan; 3 Rakuno Gakuen University, Hokkaido, Japan; 4 Tokyo Medical and Dental University Hospital of Medicine, Tokyo, Japan

## Introduction

Direct hemoperfusion with a polymyxin B immobilized column (PMX-DHP) adsorbs endotoxin and has been used for the treatment of septic shock [1]. However, the mechanisms of action behind PMX-DHP are not fully understood. Therefore, the purpose of this study was to elucidate mechanisms of action behind PMX-DHP in a rat model of cecal ligation and puncture.

## Methods

Sprague-Dawley rats were anesthetized and were mechanical ventilated after tracheostomy. The right internal carotid artery was cannulated with a catheter for continuous measurement of the arterial pressure and heart rate. The right femoral vein was cannulated with a catheter for infusion of saline (10 ml/kg/hour) during the study period. The rats were randomized into three experimental groups: cecal ligation and puncture (CLP) + dummy column (Dummy-DHP) group (*n *= 10), CLP + PMX-DHP group (*n *= 10), and sham group (*n *= 4). Four hours after CLP, Dummy-DHP or PMX-DHP was performed for 1 hour. Blood was drawn from the right internal carotid artery, perfused through PMX column or dummy column, and returned to the right femoral vein. The heart rate, mean arterial pressure, arterial blood gases, and plasma concentrations of creatinine, lactate, potassium, and cytokines (IL-6 and IL-10) were measured at baseline and at 4, 5, and 8 hours after CLP. At the completion of the experiment, the rats were killed overdose of pentobarbital. The kidney, liver, and lung were harvested, and histopathologic examinations of these organs were performed.

## Results

Hypotension and metabolic acidosis occurred in the CLP + Dummy-DHP group, whereas hemodynamics and acid-base balance were better maintained in the CLP + PMX-DHP group. Plasma concentrations of lactate, creatinine, potassium, and cytokines were significantly higher in the CLP + Dummy-DHP group than in the CLP + PMX-DHP group at 8 hours. Renal tubular cell death was observed in the CLP + Dummy-DHP group, but not in the CLP + PMX-DHP group.

## Conclusion

PMX-DHP improved hemodynamics, acid-base balance, and creatinine levels through reducing cytokines and renal tubular cell death in a rat model of cecal ligation and puncture. These findings suggest the preventive role of PMX-DHP in the development of sepsis-related acute kidney injury.
